# Chicken DDX3X Activates IFN-β via the chSTING-chIRF7-IFN-β Signaling Axis

**DOI:** 10.3389/fimmu.2019.00822

**Published:** 2019-04-17

**Authors:** Qiaona Niu, Yuqiang Cheng, Hengan Wang, Yaxian Yan, Jianhe Sun

**Affiliations:** Shanghai Key Laboratory of Veterinary Biotechnology, Key Laboratory of Urban Agriculture (South), School of Agriculture and Biology, Shanghai Jiao Tong University, Ministry of Agriculture, Shanghai, China

**Keywords:** chicken, DDX3X, STING, IFN-β, RNA virus, innate immunity

## Abstract

Asp-Glu-Ala-Asp (DEAD)-box polypeptide 3 X-linked (DDX3X) is an ATP-dependent RNA helicase, In addition to involvement of eukaryotic gene expression regulation, mammalian DDX3X has recently been found to regulate IFN-β production via the adaptor MAVS mediated cascade signaling. In our studies, we demonstrated that chicken DDX3X (chDDX3X) is also involved in the IFN-β regulation, and demonstrated that chDDX3X regulated IFN-β via an essential adaptor chicken stimulator of IFN genes (chSTING). We found that chDDX3X overexpression in DF-1 cells induced expression of IFN-β and inhibited avian influenza virus (AIV) or Newcastle disease virus (NDV) replication. Knockdown of chDDX3X decreased the production of IFN-β induced by RNA analog polyinosinic-polycytidylic acid and increased viral yield. Furthermore, chDDX3X was identified as a potential chSTING-interacting protein by co-immunoprecipitation (Co-IP) and liquid chromatography-tandem mass spectrometry (LC-MS/MS). And exogenous Co-IP in transfected cells with or without virus-stimulations further confirmed the interaction between chDDX3X and chSTING. With the gene overexpression and RNA interference studies, the chDDX3X was confirmed to be located upstream of chSTING and activate IFN-β via the chSTING-chTBK1-chIRF7-IFN-β signaling axis. In brief, our results suggest that chDDX3X is an important IFN-β mediator and is involved in RNA- and RNA virus-mediated chDDX3X-chSTING-IFN-β signaling pathway.

## Introduction

Innate immunity acts as the first line of defense against invading organisms. Innate immune responses are activated by host pattern-recognition receptors (PRRs), which recognize conserved molecules presenting in certain pathogen classes, collectively referred to pathogen-associated molecular patterns (PAMPs) (1–3). The main PRR groups that recognize viral nucleic acids are Toll-like receptors (TLRs), retinoic acid inducible gene I (RIG-I)-like receptors, which are part of the DExD/H family of RNA helicases, and cytoplasmic DNA receptors (4). Upon activation by PAMPs, PRRs recruit and activate their respective adaptors, such as MAVS, TRIF and MyD88, and trigger the activation of the transcription factors IRF3/7 and NF-κB, which result in the production of antiviral and proinflammatory cytokines, including type I interferons (IFN-α and IFN-β) (5–8).

DDX3X, as well as the well-known RNA recognition sensor RIG-I (also known as DDX58) and MDA5, belongs to the DEAD-box helicase family. This family has at least eight conserved motifs and the motif II (Asp-Glu-Ala-Asp, or DEAD) provides the family's name (9, 10). The DEAD-box proteins are involved in all steps of RNA metabolism (10–13). DDX3X is a homolog of DDX3 gene. DDX3 has two homologs, DDX3X and DDX3Y, located in chromosomes X and Y, respectively (6). DDX3X has been demonstrated to be related to a variety of cellular processes involving RNA, such as transcriptional regulation, splicing, mRNA exportation, ribosome biogenesis, and translational regulation. Recently, DDX3X has received intensive attention because several studies demonstrated that it participates in regulation of innate immune responses by functioning as the viral nucleic acid sensor or facilitating downstream signaling pathway (14, 15). Upon activation, DDX3X activates the type I interferon responses via the MAVS-TBK1/IKKε-IFN-β signaling axis and triggers the antiviral response (16). Although DDX3X has been identified in mammalian cells and its functions in the innate immunity have been partly studied (17), it has not yet been identified in birds, and its involvement in the IFN response is hitherto unknown. If chDDX3X involved in the IFN regulation, the molecular mechanism of chDDX3X in IFN activation in bird cells is unclear.

STING (also called MITA, MYPS, and ERIS) is an endoplasmic reticulum (ER) resident transmembrane protein (18). In mammalian cells, STING interacts with both MAVS and RIG-I and acts as a multifaceted IFN mediator in DNA- or RNA-triggered IFNs signaling pathways (5, 18, 19). In our previous study, we found that although RIG-I is absent in chickens, chicken MDA5 can interacts with STING to form an MDA5-STING-IFN-β pathway, which may be absent in mammals (20). Furthermore, through endogenous co-immunoprecipitation (Co-IP) and liquid chromatography-tandem mass spectrometry (LC-MS/MS), chDDX3X was found as another protein interacting with chSTING. In particular, the discovery that chDDX3X interacts with chSTING allows us to speculate that chDDX3X may participate in IFN regulation in a chSTING-dependent manner. Due to those finding, the relationship between chDDX3X and chSTING and its role will be explored in the present study.

In present study, the first bird DDX3X was cloned from chicken cells, and its function in the type I IFN signaling pathway was explored, which revealed its functions in innate immunity against RNA and RNA virus in avian. Moreover, the results elucidated that chDDX3X can interact with chSTING and activate IFN via the chDDX3X-chSTING-IFN-β axis. These results will expand our knowledge of the relationship between mammalian and birds in innate immunity, and help us improve the understanding of the biological role of DDX3X in evolution of innate immunity.

## Materials and Methods

### Cells and Viruses

Chicken embryonic fibroblast cell line DF-1 from East Lansing eggs was cultured, as described in our previous study (21), were maintained in a high-glucose Dulbecco's modified Eagle's medium (Life Technologies, Grand Island, NY) supplemented with 10% fetal bovine serum (Life Technologies), and incubated at 37°C in a 5% CO_2_ incubator. Chicken TBK1 and IRF7 gene knockout DF-1 cell lines (chTBK1^−/−^ and chIRF7^−/−^ cells) were generated in our laboratory using the CRISPR/Cas9 technique (22). The A/Chicken/Shanghai/010/2008 (H9N2) avian influenza virus (SH010 AIV) was isolated from chickens in Shanghai, China in 2008. Newcastle disease virus (NDV) strain Herts/33 was obtained from the China Institute of Veterinary Drug Control (Beijing, China).The viruses were purified, propagated, and stored as described in our previous study (23).

### Cloning and Bioinformatics Analysis of chDDX3X

Based on the predicted chicken chDDX3X sequence (XM_015301535.1) from the National Center for Biotechnology Information (NCBI), primers chDDX3X-F and chDDX3X-R (Table 1) were designed and used to amplify the chDDX3X cDNA from chicken DF-1 cells by RT-PCR. The PCR product was cloned into a pTOPO-Blunt Cloning Kit, which comes from Aidlab Biotechnologies Company (Beijing, China). The amino acid sequences of chDDX3X and DDX3X of other animals from such as human, mouse, pig, and cattle were aligned using BioEdit ClustalW multiple alignment. Phylogenetic analysis of amino acid sequences were conducted using MEGA5.0. The phylogenetic tree was constructed with DDX3X from 17 different species, including fish, birds, and mammals.

**Table 1:** PCR primers used in the study.

**Target gene**	**Purpose**	**Name**	**Sequence of oligonucleotide (5**′**-3**′**)**
chDDX3X	To obtain sequence	chDDX3X F	ATGAGTCATGTGGCGGTGGAAAATG
		chDDX3X R	TCAGTTGCCCCACCAGTCAAC
	Cloning	p3 × Flag-14-CMV Kpn I Fp3 × Flag-14-CMV BamH I R	gatagatctgatatcggtaccATGAGTCATGTGGCGGTGGA tttgtagtcagcccgggatccGTTGCCCCACCAGTCAACC
		pcDNA3.1-Flag Kpn I FpcDNA3.1-Flag BamH I R	tttaaacttaagcttggtaccATGAGTCATGTGGCGGTGGA ccacactggactagtggatccGTTGCCCCACCAGTCAACC
chSTING	Cloning	pCMV-HA Bgl II F	gaattcggtcgaccgagatctCTATGCCCCAGGACCCGT
		pCMV-HA Not I R	catgtctggatccccgcggccgcTCAGGGGCAGTCACTGCG
		pcDNA3.1-Flag Kpn I F	tttaaacttaagcttggtaccATGCCCCAGGACCCGTCA
		pcDNA3.1-Flag BamH I R	ccacactggactagtggatccGGGGCAGTCACTGCGCAG
chTBK1	Cloning	Bgl II TBK1 F	gaattcggtcgaccgagatctCTATGCAGAGCACCTCGAATTACC
		Not I TBK1 R	catgtctggatccccgcggccgcTTAGATACAGTCCACATTCCTCAGGC
chIRF7	Cloning	Bgl II IRF7 F	gaattcggtcgaccgagatctCTATGGCAGCACTGGACAGCG
		Not I IRF7 R	catgtctggatccccgcggccgcTCAGTCTGTCTGCATGTGGTATTG
chDDX3X	qRT-PCR	qDDX3X F	ATGAGGAGGCCAGGAAGTTT
		qDDX3X R	CTGAGGTTCGAAACCCATGT
chIFN-β	qRT-PCR	qIFN-β F	CCTCAACCAGATCCAGCATT
		qIFN-β R	GGATGAGGCTGTGAGAGGAG
chIRF-7	qRT-PCR	qIRF-7 F	GCCTGAAGAAGTGCAAGGTC
		qIRF-7 R	CTCTGTGCAAAACACCCTGA
chSTING	qRT-PCR	qSTING F	GGTCCTACTACATCGGCTACCTGA
		qSTING R	GGCCTGAGCTTGTTGTCCTTATCT
chPKR	qRT-PCR	qPKR F	TGCTTGACTGGAAAGGCTACT
		qPKR R	TCAGTCAAGAATAAACCATGTGTG
chMx-1	qRT-PCR	qMx-1 F	GTTTCGGACATGGGGAGTAA
		qMx-1 R	GCATACGATTTCTTCAACTTTGG
IL-1β	qRT-PCR	qIL-1β F	TCGACATCAACCAGAAGTGC
		qIL-1β R	GAGCTTGTAGCCCTTGATGC
IL-6	qRT-PCR	qIL-6 F	GACGAGGAGAAATGCCTGAC
		qIL-6 R	GACTTCAGATTGGCGAGGAG
IL-8	qRT-PCR	qIL-8 F	GCTCTGTCGCAAGGTAGGAC
		qIL-8 R	GGCCATAAGTGCCTTTACGA
chβ-actin	qRT-PCR	qβ-actin F	CAGACATCAGGGTGTGATGG
		qβ-actin R	TCAGGGGCTACTCTCAGCTC

### Plasmid Construction, Transfection, and Luciferase Reporter Assays

The chicken IFN-β (chIFN-β) promoter luciferase reporter plasmid pGL-chIFN-β-Luc was constructed as described previously (20). Using PCR and the primers described in Table 1, the Flag-tagged chDDX3X plasmids were constructed by inserting full-length chDDX3X into the *Kpn* I and *Bam*H I sites p3 × FLAG-14-CMV or pcDNA3.1-FLAG of the expression vector using a ClonExpress II One Step Cloning Kit (Vazyme, Nanjing, China). The expression plasmids pCMV-HA-chSTING, pCMV-HA-chTBK1, and pCMV-HA-chIRF7 were constructed by inserting full-length chSTING, chTBK1, and chIRF7 into the *Bgl* II and *Not* I sites of the pCMV-HA expression vector. To construct pcDNA3.1-FLAG-chSTING plasmid, we inserted full-length chSTING into the *Kpn* I and *Bam*H I sites of the pcDNA3.1-FLAG. DF-1 cells seeded in 24-well plates were transfected with the indicated plasmids using FuGENE HD (Promega, Madison, WI). The dual luciferase reporter assays were performed as the manufacturer's instructions (Promega). Briefly, cells were co-transfected with expression plasmids or the empty vector (0.5μg/well), the firefly reporter plasmid pGL-chIFN-β-Luc (0.1 μg/well), and the pRL-TK plasmids expressing Renilla luciferase (0.05μg/well) used as the internal reference. All reporter assays were repeated at least three times.

### Co-immunoprecipitation Assay

For exogenous Co-IP experiments, DF-1 or HEK 293T cells were seeded in 60-mm dishes (1 × 10^7^ cells/dish) overnight and co-transfected with 4 μg of empty plasmids and various expression plasmids, respectively. At 48 h post-transfection, the medium was removed carefully, and the cell was washed twice with ice-cold PBS. For Co-IP with viral stimulation experiments, DF-1 cells were transfected with 6 μg of empty plasmids or expression plasmids. At 24 h post-transfection, cells were stimulated with SH010 AIV or Herts/33 NDV. At 8 h after infection, the cells were harvested as above. Then, cells were lysed with 500 μL of RIPA lysis buffer (50 mM Tris [pH 7.4], 150 mM NaCl, 1% NP-40, 0.25% sodium deoxycholate, sodium orthovanadate, sodium fluoride, EDTA, and leupeptin) (Beyotime, Shanghai, China) containing protease cocktail (Yeasen, Shanghai, China). Lysates were centrifuged at 14,000 × *g* for 5 min. The supernatant was transferred to a new tube and precipitated with 20 μL of anti-Flag or anti-HA affinity gel (Biotool, Houston, TX) for 2 h at 4°C. The affinity gel was washed with cold TBS four times and mixed with TBS and 5 × sodium dodecyl sulfate (SDS) loading buffer (Yeasen), and then were boiled for 8 min. The cell lysates were also mixed with 5 × SDS loading buffer and boiled. Proteins eluted from the beads and cell lysates were separated by SDS-PAGE and analyzed by Western blotting using the indicated antibodies.

### Western Blot Analysis

For Western blots, the samples resolved by SDS-PAGE were transferred onto polyvinylidene fluoride (PVDF) membranes. Non-specific protein interactions were blocked with 5% skim milk in TBST buffer (TBS buffer containing 0.05% Tween-20) for 1 h at room temperature. The membranes were incubated in TBST buffer for 2 h at room temperature with the appropriate primary antibodies: anti-chDDX3X and anti-chIRF7 polyclonal antibodies were obtained by using the recombinant proteins to produce mouse polyclonal mouse antiserum by immunizing mice; anti-TBK1 (ABGENT, Shanghai, China); anti-phospho-TBK1 (Cell Signaling, MA, USA); anti-HA and anti-Flag (Sigma, MO, USA); anti-HA and anti-Flag beads (Biotool, Houston, TX); and anti-β-tubulin (TransGen, Beijing, China). The blotted membranes were washed three times in TBST buffer and then incubated with 1:5,000 dilutions of horseradish peroxidase-conjugated secondary antibodies in TBST buffer for 1 h. The proteins were visualized using bioluminescence reagents (Pierce, Rockford, IL) in accordance to the manufacturer-specified protocol. The images were collected with a Tanon 5200 imaging system (Tanon, Shanghai, China).

### Expression of Short Hairpin RNA

Specific chDDX3X gene knockdown in DF-1 cells was conducted via pGPU6-based silencing (GenePharma, Shanghai, China). Two RNA interference (RNAi) target sequences (Table 2) against chDDX3X, and a negative control sequence, which is not present in human or chicken genome databases, were designed. Two complementary short hairpin RNA (shRNA) template oligonucleotides were synthesized according to the designed RNAi sequences against chDDX3X and the negative control. Then, the complementary oligonucleotides were annealed and inserted into the shRNA expression vector pGPU6-neo. The recombinant shRNA plasmids were named shDDX3X-1, shDDX3X-2, and shNC, respectively. The shSTING, shIRF-7, shTBK1-1, and shTBK1-2 plasmids were constructed in our previous study (20). For silencing, DF-1 cells in 12- or 24-well plates were transfected with shRNA at 1 μg/well or 500 ng/well using FuGENE HD (Promega). At 48 h post-transfection, the cells were used for subsequent experiments.

**Table 2:** RNAi target sequences.

**Target gene**	**Name**	**Sequence of oligonucleotide (5**′**-3**′**)**	**Accession no**.
chDDX3X	shDDX3X-1	GCAGAGACAAGGATGCATACA	MH119574
	shDDX3X-2	GCAATTGTCCTCCACATATTG	
Negative control	shNC	GTTCTCCGAACGTGTCACGT	

### RNA Extraction and Quantitative Real-Time PCR

Total RNA was extracted from the tissues or cells using an HP Total RNA kit (Omega). RNAs were reverse transcribed to cDNA using a cDNA synthesis kit (Vazyme, Nanjing, China), and quantitative real-time PCR (qRT-PCR) was conducted using the indicated primers (Table 1) on an ABI 7500 real-time PCR system. The qRT-PCR was performed according to the manufacturer's instructions using ChamQTM SYBR® qPCR Master Mix (Vazyme). The PCR conditions were as follows: 1 cycle at 95°C for 30 s, 40 cycles of denaturation at 95°C for 10 s, 60°C for 30 s, followed by a dissociation curve analysis step at 95°C for 15 s, 60°C for 60 s, and 95°C for 15 s to verify the amplification of single and specific products. The relative expression levels for the tested mRNAs were determined using β-actin as an internal reference using the comparative Ct ($2^{-\Delta \Delta_{\text{CT}}}$) method.

### shRNA-Mediated Signal Disruption Experiment

For the signal disruption experiment mediated by gene overexpression, DF-1 cells in 24-well plates were co-transfected with the dual luciferase reporter plasmids (pGL-chIFN-β-Luc and pRL-TK) and shRNA plasmids. At 24 h post-transfection, the cells were transfected again with the indicated expression plasmids. At 24 h after the second transfection, the cells were harvested to detect luciferase activity. In poly(I:C)-stimulus experiments, DF-1 cells were co-transfected with luciferase reporter plasmids and shRNA. At 42 h post-transfection, cells were transfected again with 0.1 μg/mL poly(I:C) (Invivogen, San Diego, CA). At 6 h post-transfection of the poly(I:C), the cells were harvested to detect luciferase activity.

### Virus Stimulation

For endogenous chDDX3X respond to viral infections, DF-1 cells were stimulated with SH010 AIV) or Herts/33 NDV at a multiplicity of infection (MOI) of 0.1. At different time points after infection, the cells were harvested to detect by qPCR and western blot analysis. For exogenous chDDX3X respond to viral infections, DF-1 cells were transfected with Flag-chDDX3X plasmid or empty vector. After 36 h, the transfected cells were washed and infected with SH010 AIV or Herts/33 NDV(0.1 MOI). At different time points after infection, the supernatant were harvested for measuring viral titers by a 50% tissue culture infective dose (TCID_50_). For viral stimulation experiments in chDDX3X-knockdown cells, RNAi plasmids of chDDX3X were transfected into DF-1 cells. After 36 h, the cells were washed and infected with SH010 AIV or Herts/33NDV (0.1 MOI). At different time points post-infection, the supernatants were harvested to measure viral titers by the standard TCID_50_ method. For poly(I:C) stimulation experiments in chDDX3X-knockdown cells, DF-1 cells were also co-transfected luciferase reporter plasmids with RNAi plasmids. At 48 h after transfection, cells were transfected again with poly(I:C). After 6 h, cells were harvested for detection of luciferase activity.

### Statistical Analysis

Data were expressed as means ± standard deviations. Significance was determined with the two-tailed independent Student's *t*-test or one-way analysis of variance (ANOVA) (^*^p < 0.05, ^**^p < 0.01).

## Results

### Chicken DDX3X Is a STING-Interacting Protein

**Figure 1:**
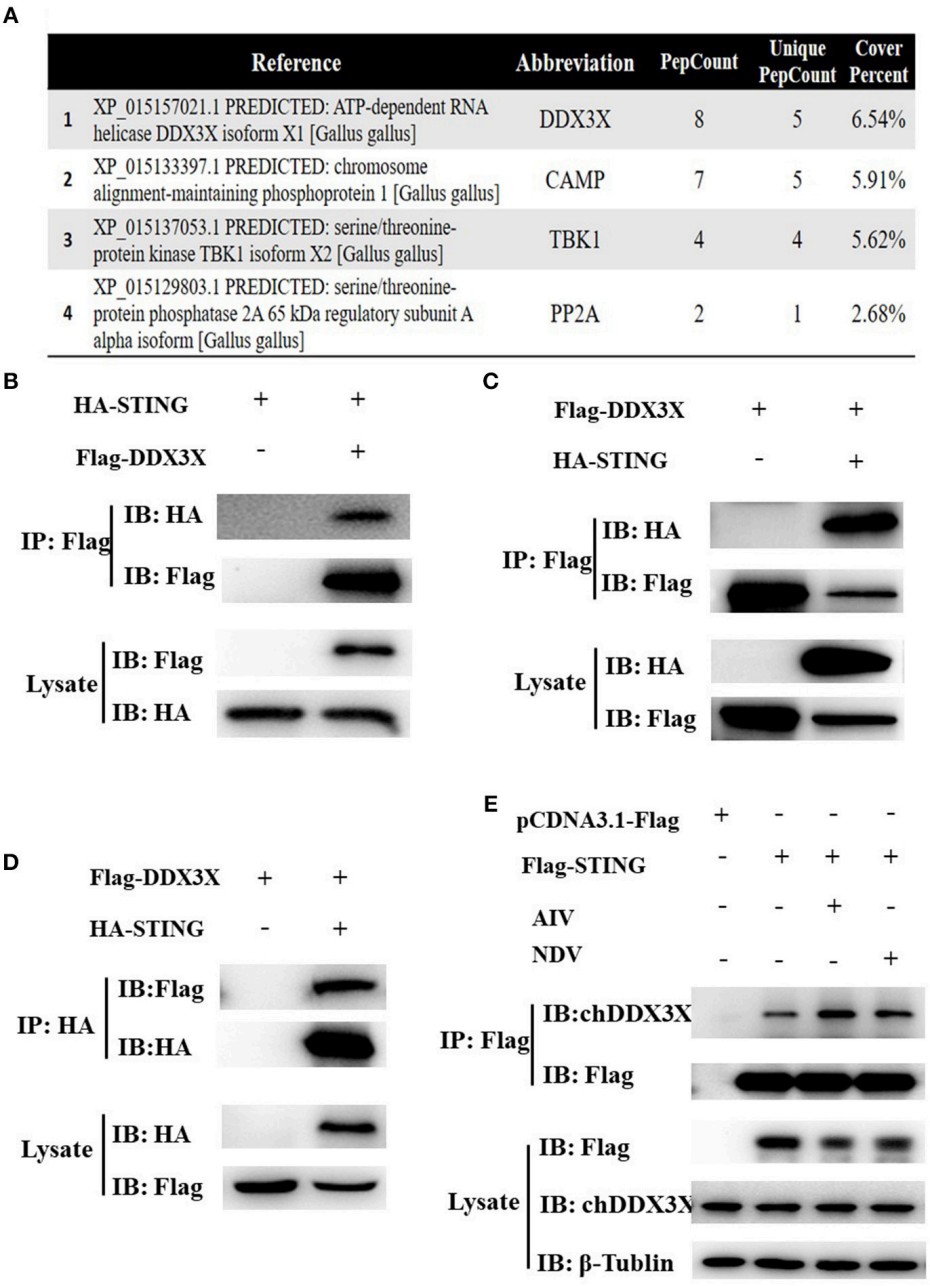
Identification of the interaction between chSTING and chDDX3X. **(A)** A partial list of the chSTING-interacting proteins obtained using liquid chromatography-tandem mass spectrometry (LC-MS/MS). **(B–D)** chSTING-HA was associated with chDDX3X-Flag in transfected cells. DF-1 cells or HEK293T cells were co-transfected with pcDNA3.1-chDDX3X-Flag, pCMV-HA-chSTING and empty vectors. At 48 h after transfection, the HEK 293T cells lysates were precipitated with an anti-Flag affinity gel **(B)**, the DF-1 cells lysates were precipitated with an anti-Flag or anti-HA affinity gel **(C,D)** and further detected using Western blot analysis with anti-Flag and anti-HA antibodies (the first two lanes). The expression of the transfected proteins was analyzed using immunoblotting with anti-Flag and anti-HA (third and bottom lanes). **(E)** chSTING-Flag was associated with chDDX3X in transfected cells. DF-1 cells were transfected with pcDNA3.1-chSTING-Flag or empty vectors. At 24 h post-transfection, cells were stimulated with SH010 AIV or Herts/33NDV. At 8 h after infection, the cells lysates were precipitated with an anti-Flag affinity gel, and further detected using Western blot analysis with chDDX3X and anti-Flag antibodies (the first two lanes). The expression of the transfected proteins was analyzed using immunoblotting with anti-Flag, chDDX3X, and β-Tubulin (third, fourth, and bottom lanes).

In our previous study on the regulatory mechanism of chSTING in the induction of IFN-β expression, chDDX3X was identified as a potential chSTING-interacting protein by endogenous Co-immunoprecipitation (Co-IP) and LC-MS/MS (Figure 1A). In this study, exogenous Co-IP was performed to further confirm the interaction between chDDX3X-Flag and chSTING-HA. The results revealed that anti-Flag mAb-immunoprecipitated protein complexes were also recognized by anti-HA pAb in HEK 293T cells (Figure 1B) or in DF-1 cells (Figure 1C). In a reverse assay, anti-HA mAb-immunoprecipitated complexes were recognized by anti-Flag mAbs in DF-1 cells (Figure 1D). The Co-IP with viral stimulation was also performed to further confirm the interaction between chSTING-Flag and endogenous chDDX3X. We found that the anti-Flag mAb-immunoprecipitated protein complexes were also recognized by chDDX3X mAb and the interaction between chSTING and chDDX3X was strengthened with viral stimulation in DF-1 cells (Figure 1E).These results confirmed that chDDX3X interacts with chSTING.

### Cloning and Sequence Analysis of chDDX3X

**Figure 2:**
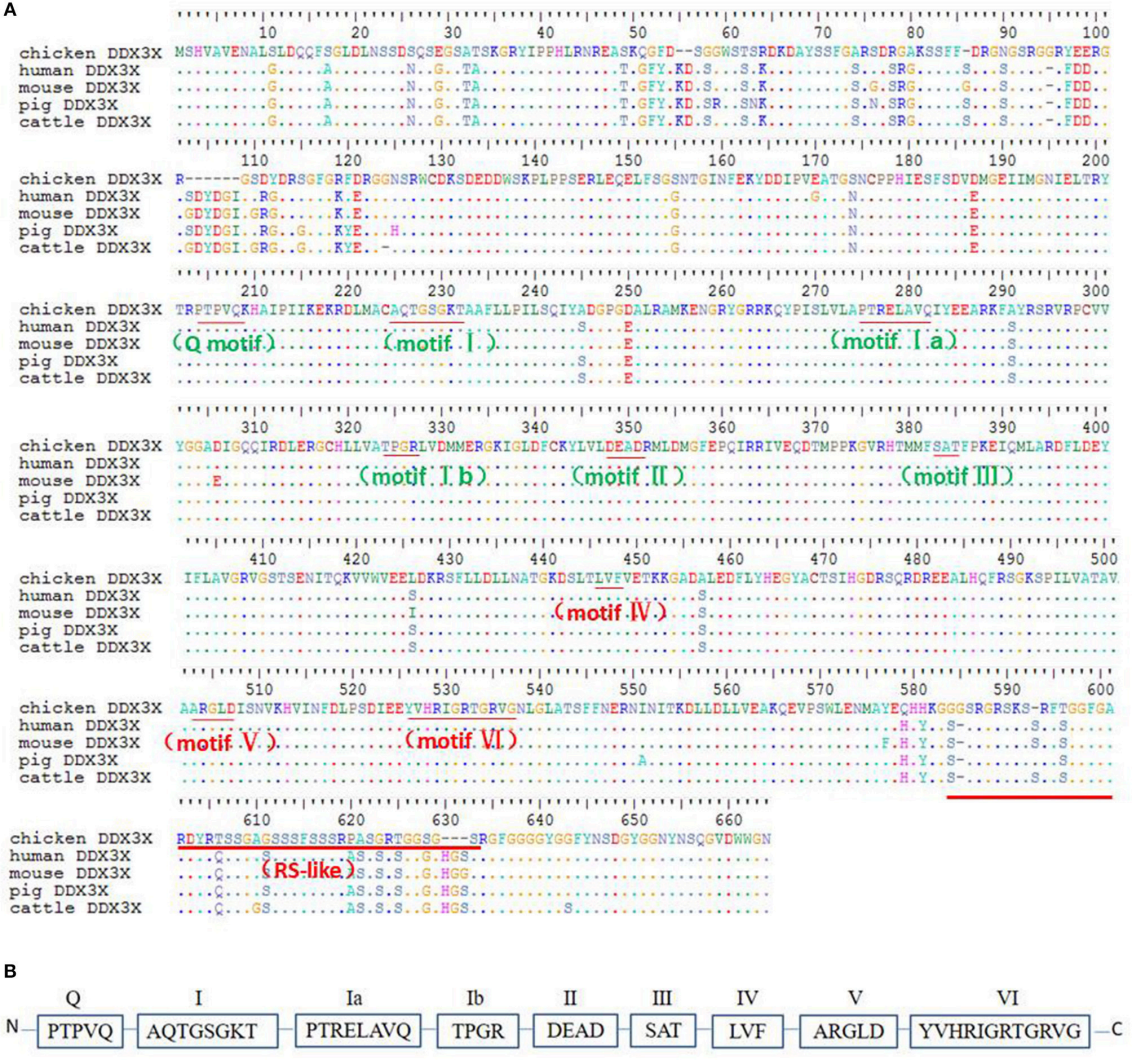
**(A)** Alignment of the amino acid sequence of chicken DDX3X with that of other animal DDX3X proteins from human, mouse, pig, and cattle. Alignment was performed using BioEdit ClustalW multiple alignment (BioEdit, UK). Spots indicate amino acid identity. Motifs are in bold. **(B)** Schematic representation of the conserved helicase boxes in DDX3X along with their sequences.

To better understand chDDX3X, it was cloned and studied using bioinformatics analysis. The nucleotide sequences of chDDX3X were deposited to GenBank under the accession number MH119574. The full-length cDNA of chDDX3X contains 1,956 bp and encodes 632 amino acid residues (Figure 2A). Multiple sequence alignment showed that the amino acid sequence of chDDX3X showed 93.1, 92.8, 92.1, and 92.4% identity to the DDX3X gene in human (*Homo sapiens*, BAG70034.1), mouse (*Mus musculus*, NP_034158.1), pig (*Sus scrofa*, NP_001233132.1), and cattle (*Bos taurus*, NP_001179891.1), respectively. We also found that the N-terminal or the arginine-serine-rich (RS) domain in the C-terminal domain of chDDX3X is poorly conserved, and the catalytic helicase core domain of chDDX3X, which is composed of helicase motifs (Q, I, II, III, IV, V, and VI) is highly conserved when compared with DDX3X in the four mammalian cells (Figures 2A,B).

**Figure 3:**
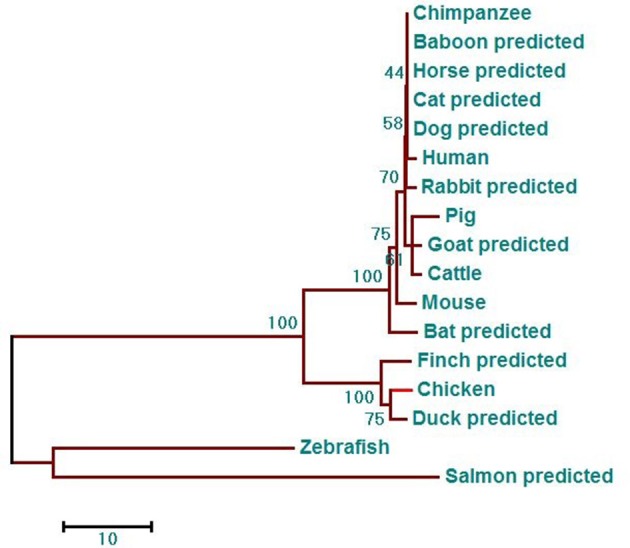
DDX3X phylogenetic tree. The phylogenetic tree is based on the amino acid sequence of chDDX3X and other known or predicted sequences of DDX3X. The identified or predicted DDX3X molecules on the phylogenetic tree are sequences from different species available from the NCBI. The phylogenetic tree was constructed using MEGA 5.0 software. The number in the phylogenetic tree represents the bootstrap value. Sequences were obtained from GenBank entries with the accession numbers BAG70034.1 (human), NP_034158.1 (mouse), NP_001233132.1 (pig), NP_001179891.1 (cattle), XP_016798881.1 (chimpanzee, predicted), XP_021788254.1 (baboon, predicted), XP_013832038.1 (goat, predicted), XP_014584362.1 (horse, predicted), XP_008270730.1 (rabbit, predicted), XP_005641313.1 (dog, predicted), XP_005880938.1 (bat, predicted), XP_004000461.1 (cat, predicted), XP_021123193.1 (mallard, predicted), XP_021123193.1 (medium ground-finch, predicted), NP_571016.2 (zebrafish, predicted), and XP_014019981.1 (salmon, predicted).

Phylogenetic analysis showed that the phylogenetic tree has two major branches (Figure 3). The DDX3X protein sequences from birds, including chicken, finch (predicted), and duck (predicted) belong to the same subgroup as the DDX3X from mammals, including humans, mice, rabbits (predicted), chimpanzees (predicted), baboons (predicted), cows (predicted), horses (predicted), goats (predicted), dogs (predicted), pigs (predicted), bats (predicted), and cats (predicted). Fish DDX3X sequences belong to the other group. The results showed that chicken DDX3X is most closely related to DDX3X of other bird species, followed by mammals, and the relationship with fish DDX3X is the most alienated.

### Chicken DDX3X Involved in the Regulation of IFN-β, Pro-Inflammatory Cytokines, and Antiviral Molecules

**Figure 4:**
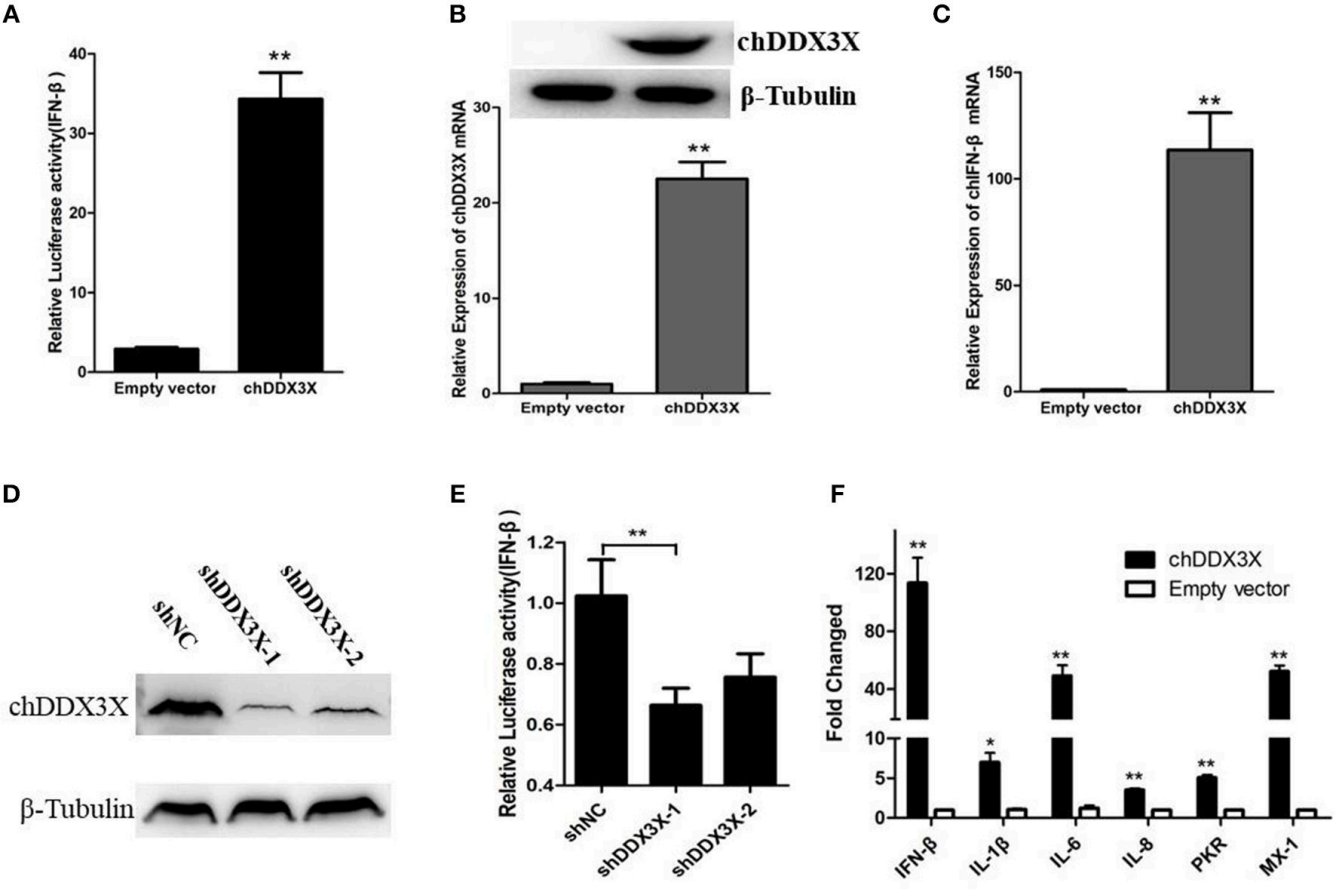
Chicken DDX3X is involved in the regulation of IFN-β expression and induces the expression of proinflammatory cytokines and antiviral molecules. **(A–C)** Overexpression of chDDX3X induced the production of IFN-β. **(A)** DF-1 cells were co-transfected with the chIFN-β luciferase reporter plasmids (pGL-chIFN-β-Luc and pRL-TK) and p3 × FLAG-14-chDDX3X or p3 × FLAG-14. Luciferase assays were performed after 24 h co-transfection. **(B,C)** DF-1 cells were transfected with p3 × FLAG-14-chDDX3X or p3 × FLAG-14, and then chDDX3X **(B)**, chIFN-β **(C)** mRNAs or proteins were analyzed by qRT-PCR or Western blotting. **(D,E)** Knockdown of chDDX3X blocks IFN-β induction. **(D)** DF-1 cells were incubated overnight in six-well plates and co-transfected with expressing plasmid p3 × FLAG-14-chDDX3X together with RNAi plasmids (shDDX3X-1, shDDX3X-2 or shNC). After 48 h, the expression levels of tagged chDDX3X were quantified by Western blotting and immunodetection using anti-Flag Ab. The β-tubulin level was determined with anti-β-tubulin mAbs for normalization. **(E)** DF-1 cells were co-transfected with the chIFN-β luciferase reporter plasmids with the RNAi plasmids of chDDX3X. At 48 h post-transfection, the cells were harvested for luciferase activity assays. **(F)** DF-1 cells respond to chDDX3X overexpression. Relative mRNA levels of ISGs (PKR and Mx-1) and proinflammatory cytokines (IFN-β, IL-1b, IL-6, and IL-8) analyzed by qRT-PCR after chDDX3X overexpression. DF-1 cells were transfected with p3 × FLAG-14-chDDX3X or p3 × FLAG-14. After 36 h, qRT-PCR was performed. Error bars represent standard deviations. The difference between the experimental and control groups was *p < 0.05 or **p < 0.01.

DDX3X proteins have been identified as a mediator of IFN-β in mammalian cells (17). To investigate whether chDDX3X induce IFN-β production, DF-1 cells were co-transfected with chDDX3X expression plasmids and chicken IFN-β luciferase reporter plasmids. We found that overexpression of chDDX3X in DF-1 cells activated the IFN-β promoter (Figure 4A). Endogenous IFN-β mRNAs were also analyzed after stimulation with Flag-chDDX3X or empty vector. The results indicated that IFN-β mRNA was significantly induced in DF-1 cells that had been transiently transfected with chDDX3X (Figures 4B,C).

To further investigate chDDX3X is involved in IFN-β signaling pathway in chicken cells, we constructed RNAi plasmids of chDDX3X (shDDX3X-1, shDDX3X-2, and shNC). To confirm specific target knockdown, DF-1 cells were co-transfected with the expression plasmids and shRNA plasmids. At 48 h post-transfection, the cells were harvested to Western blot analysis. The results revealed that both shDDX3X-1 and shDDX3X-2 substantially reduced the expression of chDDX3X, and is the specific knockdown (Figure 4D). To determine whether chDDX3X knockdown blocks IFN-β induction, DF-1 cells were co-transfected with the IFN-β reporter system plasmids and RNAi plasmids (shDDX3X-1, shDDX3X-2, or shNC). At 48 h after transfection, luciferase reporter assays were performed. Knockdown of chDDX3X expression resulted in reduced activity from the IFN-β promoter even without any stimulus (Figure 4E). These results showed that silencing chDDX3X significantly reduced IFN-β promoter activation in DF-1 cells.

To further understand immune induction by chDDX3X, we examined the mRNAs of the ISGs (PKR and Mx-1) and the proinflammatory cytokines (IL-1β, IL-6, and IL-8) using real-time RT-PCR. The results showed that the mRNA levels of all examined genes were significantly increased with chDDX3X overexpression (Figure 4F). Mx-1, PKR, IL-1β, IL-6, and IL-8 expression levels were 52.1-fold, 5.0-fold, 5.75-fold, 78.29-fold and 7.40-fold higher than their corresponding levels in the empty vector transfected controls, respectively. Thus, chDDX3X overexpression induced the expression of antivirus molecules and proinflammatory cytokines as well as the expression of IFN-β.These results indicated that chDDX3X is involved in the regulation of IFN-β, proinflammatory cytokines, and antiviral molecules expression.

### Responses of chDDX3X to Viral RNA

**Figure 5:**
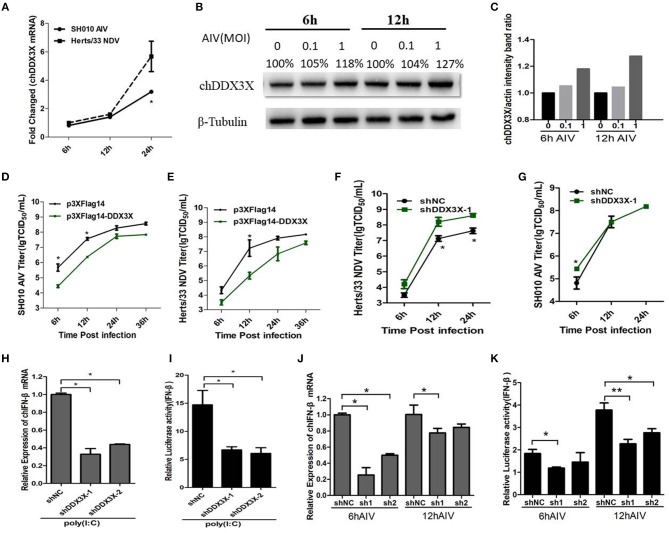
DF-1 cells respond to infection with the dsRNA mimic poly(I:C), SH010 AIV or Herts/33 NDV. **(A–C)** Chicken DDX3X was upregulated in the SH010 AIV or Herts/33NDV-infected DF-1 cells. DF-1 cells were infected with SH010 AIV or Herts/33 NDV at an MOI of 0.1. At 6, 12 and 24 h post-infection, the cells were collected for qPCR **(A)** and Western blotting analysis **(B)**. For quantification of protein levels of Figure 5B, the density of bands was analyzed with image J software **(C)**. **(D–G)** Overexpression of chDDX3X inhibits viral yield, while knockdown of chDDX3X increases viral yield. DF-1 cells were transfected with p3 × FLAG-14-chDDX3X or p3 × FLAG-14. After 36 h, cells were infected by SH010 AIV **(D)** or Herts/33 NDV **(E)** at 0.1 MOI. The supernatants were collected at the indicated time points and analyzed for TCID_50_ titers. DF-1 cells were transfected with shDDX3X-1 or shNC. At 36 h after transfection, the cells were infected with Herts/33 NDV **(F)** or SH010 AIV **(G)** (MOI = 0.1). At 6, 12, and 24 h post-infection, the supernatants were used to measure the viral titers by a standard TCID50 method. Results are for three independent experiments. Error bars are SDs. **(H–K)** Chicken DDX3X knockdown inhibits poly (I:C)-, SH010 AIV-stimulated IFN-β mRNA levels and IFN-β promoter activation. DF-1 cells were transfected RNAi plasmids of chDDX3X (shDDX3X-1, shDDX3X-2, or shNC). Or cells were co-transfected with the chIFN-β luciferase reporter plasmids and RNAi plasmids of chDDX3X. At 42 h post-transfection, the cells were transfected again with poly(I:C) **(H,I)** or infected with SH010 AIV **(J,K)**. At the indicated time points, the cells were harvested to measure IFN-β levels of mRNA and luciferase activity. Error bars represent standard deviations. The difference between the experimental and control groups was *p < 0.05 or **p < 0.01.

The mammalian DDX3X is involved in type I IFN-mediated antiviral innate immune response. However, the function of chDDX3X in the antiviral response remains unknown. To determine whether chDDX3X could respond to the influenza virus and induce an antiviral response, we firstly analyzed the expression of chDDX3X in DF-1 cells following infection with the SH010 AIV. The results showed that both the mRNA and protein levels of chDDX3X were upregulated at the early stages of viral infection (Figures 5A–C). Then, the chDDX3X-overexpressing and normal DF-1 cells were inoculated with SH010 AIV or Herts/33 NDV and the TCID_50_ was determined. The results showed that viral titers of chDDX3X-overexpressing DF-1 cells were lower than those of the control cells at all tested time points (Figures 5D,E). This result suggests that chDDX3X overexpression in DF-1 cells suppresses AIV and NDV viral replication.

To determine whether endogenous chDDX3X was required for virus-trigged innate immune responses, the chDDX3X-knockdown cells were incubated with SH010 AIV or Herts/33 NDV, and TCID_50_ was performed. We found that the viral titers of Herts/33 NDV were higher in the shDDX3X group compared with the shNC group (Figure 5F), although SH010 AIV group showed a smaller difference at an early stage of viral infection (Figure 5G). This suggests that knockdown of chDDX3X blocked virus stimulation and increases viral yield. Furthermore, the chDDX3X-knockdown cells or chDDX3X-knockdown cells transfected with luciferase reporter plasmids were incubated with poly(I:C) or SH010 AIV, and then the IFN-β mRNA levels and luciferase activity was measured. The results demonstrated that knockdown of chDDX3X expression resulted in reduced the expression levels of IFN-β mRNA and the activity of IFN-β promoter after poly(I:C) stimulation (Figures 5H,I), and the mRNA levels of IFN-β and the activation of the IFN-β promoters were inhibited in chDDX3X knockdown groups compared with control groups at an early stage of SH010 AIV infection (Figures 5J,K). These data showed that SH010 AIV or Herts/33 NDV induced IFN-β via a chDDX3X-dependent pathway. All data suggested that chDDX3X may play an essential role in type I IFN-mediated antiviral innate immune response.

### Chicken DDX3X Mediates IFN-β Induction Via the chSTING-chTBK1-chIRF7-IFN-β Signaling Axis

In the above experiments, we demonstrated that chDDX3X takes a part in antiviral innate immune response by regulating IFN-β production. However, the mechanism of how chDDX3X regulates IFN expression is unknown. Given that chDDX3X was identified as a chSTING-interacting protein and chSTING regulates IFN-β production via the chSTING-chTBK1-chIRF7-IFN-β signaling pathway, we speculated that chDDX3X may activate the IFN expression dependent on chSTING and the chSTING-mediated IFN production signaling pathway.

**Figure 6:**
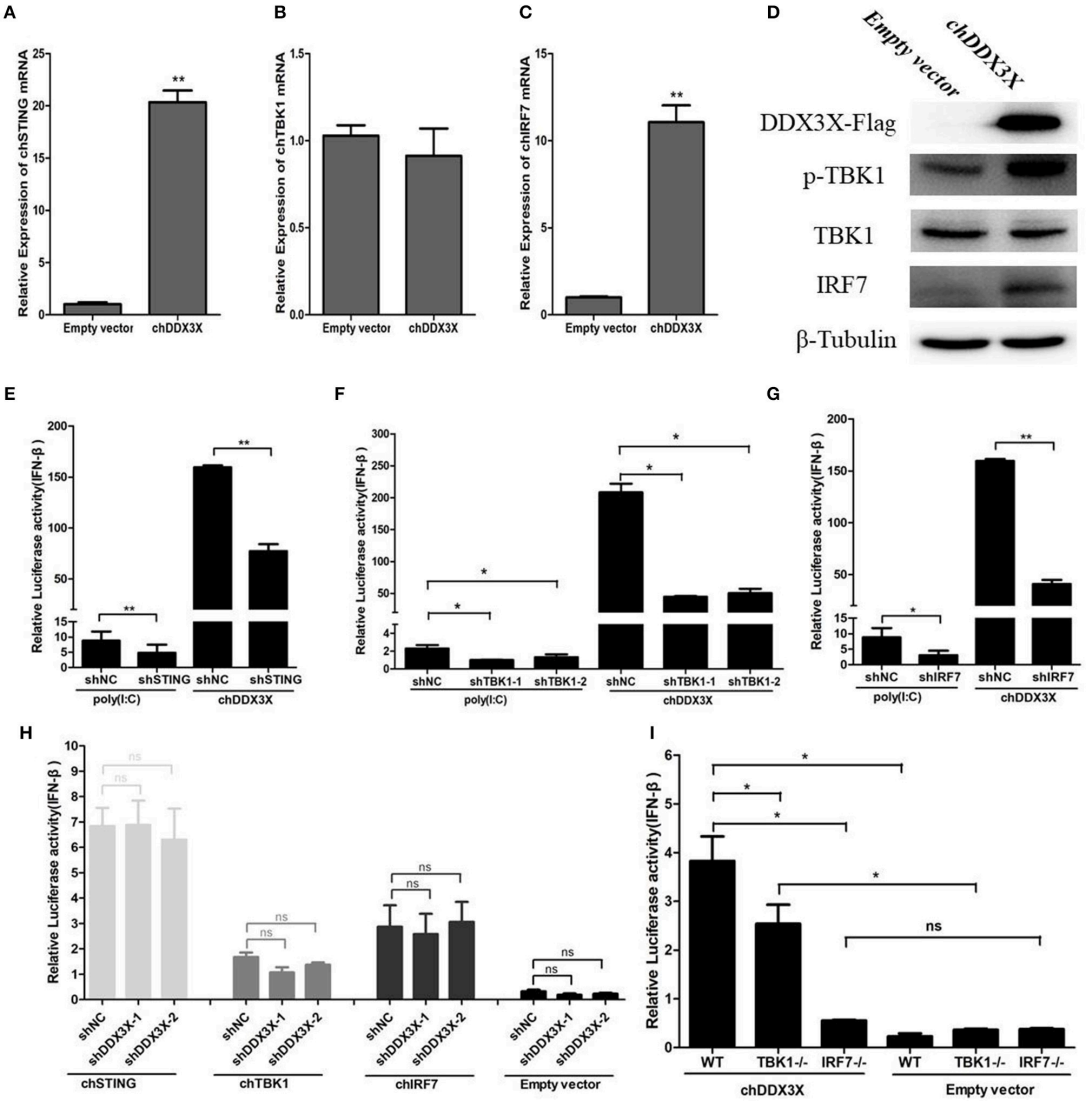
Chicken DDX3X mediates type I IFN induction via chSTING, chTBK1, and chIRF-7. **(A–C)** Relative mRNA levels of chSTING **(A)**, chTBK1 **(B)**, and chIRF7 **(C)**. DF-1 cells were transfected with p3 × FLAG-14-chDDX3X or p3 × FLAG-14. After 36 h, qRT-PCR tests were performed. **(D)** The relative protein levels of p-chTBK1, chTBK1, and chIRF7. DF-1 cells were transfected with chDDX3X or empty vector. At 36 h post-transfection, the cells were harvested for Western blot analysis assays. **(E–G)** Effect of shRNA-mediated silencing of chSTING **(G)**, chTBK1 **(H)**, and chIRF-7 **(I)** on IFN-β promoter activation induced by overexpression of chDDX3X or poly(I:C) stimulation. With chDDX3X stimulus, DF-1 cells were co-transfected with the luciferase reporter system plasmids, p3 × FLAG-14-chDDX3X and RNAi plasmids (shSTING, shTBK1-1, shTBK1-2, shIRF7, or shNC) and luciferase reporter assays were performed 48 h after transfection. With poly(I:C) stimulus, DF-1 cells were co-transfected with the reporter system plasmids and RNAi plasmids, at 42 h after transfection, the cells were transfected with poly(I:C) and luciferase reporter assays were performed. **(H)** Effect of silencing chDDX3X on IFN-β promoter activity induced by chSTING, chTBK1 or chIRF-7 overexpression. DF-1 cells were co-transfected with reporter system plasmids and RNAi plasmids (shDDX3X-1, shDDX3X-2, and shNC), At 24 h post-transfection, cells were transfected again with expression plasmids (pCMV-HA-chSTNG, pCMV-HA-chTBK1, pCMV-HA-chIRF7, or pCMV-HA). After 48 h, the cells were harvested to detect luciferase activity. **(I)** The ability of chIRF7^−/−^ and chTBK1^−/−^ cell lines to activate IFN-β promoter. DF-1 cells, chIRF7^−/−^ cells and chTBK1^−/−^ cells were co-transfected with the IFN-β reporter system plasmids and p3 × FLAG-14-chDDX3X or p3 × FLAG-14. After 24 h, luciferase reporter assays were performed. Error bars represent standard deviations. The difference between the experimental and control groups was *p < 0.05 or **p < 0.01.

The qPCR was used to detect the mRNA levels of chSTING and the genes downstream of chSTING, including chTBK1 and chIRF7, after chDDX3X overexpression. The result indicated that the chSTING and chIRF-7 mRNA expression levels increased by 20.4- and 11.1-folds, respectively (Figures 6A,B), while chTBK1 expression only changed slightly (Figure 6C). The results of the Western blot analysis revealed that chIRF7 proteins were also upregulated following chDDX3X stimulation (Figure 6D). The background expression levels of chTBK1 showed no difference between the stimulated and the control group (Figure 6D). However, the phosphorylation level of chTBK1, which is a prerequisite for the activation of the chSTING-mediated IFN pathway, was elevated in chDDX3X-stimulated cells (Figure 6D). These results suggest that chDDX3X, chSTING, chTBK1, and chIRF7 are closely related to the signaling pathway that induces IFN-β production.

To confirm that chDDX3X-mediated activation of IFN production is dependent on chSTING and chSTING-mediated signaling, the shRNA-mediated RNA interference experiments were conducted. The shRNA targeting of chSTING substantially decreased IFN-β promoter activation in cells stimulated with chDDX3X or poly(I:C) (Figure 6E). Knockdown of chTBK1 and chIRF7 expression also resulted in reduced IFN-β promoter activity after stimulation with chDDX3X or poly(I:C) (Figures 6F,G). However, silencing chDDX3X did not affect the IFN-β promoter activity induced by chSTING, chTBK1, and chIRF-7 overexpression. These finding indicated that chDDX3X regulates IFN-β through chSTING dependent pathway and functions upstream of chSTING mediated signaling (Figure 6H).

To further demonstrate that chDDX3X activates IFN production via chSTING mediated signaling, we generated chTBK1 and chIRF7 genes knockout DF-1 cell lines, which are downstream genes of chSTING, using the CRISPR/Cas9 system. Knockout and wild type DF-1 cells were transfected with chDDX3X-Flag or empty plasmids. The results showed that the IFN-β induced by chDDX3X overexpression was completely abrogated in chIRF7^−/−^ cells and markedly decreased in chTBK1^−/−^ cells (Figure 6I). Together, the findings of our study demonstrate that chDDX3X mediates IFN-β induction via the chSTING-chTBK1-chIRF7-IFN-β signaling axis.

## Discussion

Through Co-IP and LC-MS/MS, and Co-IP with or without virus stimulations experiments in DF-1 cells, we identified chDDX3X as a chSTING-interacting protein (Figures 1A–E). As chSTING was found to be an important mediator of IFN signaling in chickens, we speculated that chDDX3X may be an IFN regulator associated with chSTING.

First, we cloned and analyzed the chDDX3X gene from chickens. We found that amino acid sequence conservation of chDDX3X is high between chickens and mammals. To some extent, this shows that the function of chicken DDX3X may be similar to that of mammalian DDX3X. However, there was a significant difference in the amino acids at the N-terminal domain and arginine-serine-rich (RS) domain at the C-terminal domain between the chicken and mammalian DDX3X genes (Figure 2). Further studies are required to investigate the impact of these differences on the function of chDDX3X. Phylogenetic analysis was carried out to illustrate the evolutionary relationship between birds, mammals, and fishes of the DDX3X gene (Figure 3). DDX3X is involved in the innate immune response against viral infections in mammalian cells (17, 24). Structurally, chDDX3X was homologous to its mammalian. As such, we speculated that chDDX3X may participate in the innate immune response. Overexpression experiment revealed that overexpression of chDDX3X strongly induced the expression of IFN-β (Figures 4A–C). On the other hand, chDDX3X knockdown by shRNA blocked the ability of DF-1 cells to induce IFN-β production (Figures 4D,E). These results indicated that chDDX3X plays a role in regulating IFN-β expression, which is consistent with findings in mammalian cells (24). In addition, chDDX3X overexpression also induced high expression of proinflammatory cytokines (Figure 4F), indicating that chDDX3X has additional roles apart from regulating IFN expression. Currently, studies on the DDX3X gene primarily focus on its functions in type I IFN-dependent innate immunity (15). The functions of the proinflammatory cytokines induced by DDX3X remain unclear.

Mammalian DDX3X plays an essential role in the antiviral innate immune response (17). In this study, in SH010AIV-infected chicken cells, the chDDX3X mRNA and protein level were upregulated compared with uninfected cells (Figures 5A–C). Upregulation is an important strategy by which some immune molecules respond to viral infection. The AIV-induced chDDX3X upregulation observed in this study is evidence for the role of chDDX3X and its antiviral effect. Notably, we also found that chDDX3X overexpression in DF-1 cells inhibited SH010 AIV or Herts/33 NDV viral replication (Figures 5D,E), while knockdown of endogenous chDDX3X increased the viral yield of NDV yield in DF-1 cells (Figure 5F). However, knockdown of chDDX3X only enhances virus replication at 6 h post-infection (Figure 5G). We speculate that it may because influenza NS1 or some other proteins interference IFN induction induced by chDDX3X at 12 and 24 h post-infection, hence no differences were observed between the chDDX3X knockdown and WT group. These results further indicated that chDDX3X could respond to the virus stimulus and participate in the antiviral response. We also found that chDDX3X knockdown blocked the dsRNA mimic poly (I:C)- or virus-stimulated IFN-β (Figures 5H–K). This indicated that chDDX3X is required for IFN-β production induced by poly(I:C) or AIV. This implied that RNA virus induced IFN-β production via chDDX3X and chDDX3X has an anti-RNA virus function. H9N2 AIV has received considerable attention as many researchers have proposed that poultry carrying this virus are incubators for novel human AIVs (25); therefore, we choose H9N2 AIV as a model virus in virus stimulation assays. These data demonstrated that chDDX3X is a critical component of the RNA virus-triggered IFN-β activation pathway and is involved in immune responses against H9N2 AIV in chicken cells.

The above mentioned studies have defined chDDX3X as an IFN activator that participates in the antiviral innate immunity; however, it is not clear how chDDX3X activates IFN signaling. As chSTING has been identified as a chDDX3X-interacting protein, we are interested to know whether chDDX3X activates IFN via chSTING signaling. In chDDX3X-overexpression cells, the chSTING and chIRF7 mRNA expression levels were significantly improved, when the p-TBK1 and chIRF7 proteins were also upregulated (Figures 6A–D). In shRNA-mediated signal interference experiments, chSTING, chTBK1, chIRF7 knockdown by shRNA substantially reduced IFN-β promoter activity induced chDDX3X overexpression, while knockdown of chDDX3X expression have no affect with the IFN-β induced by chSTING, chTBK1 and chIRF-7 (Figures 6E–H). In addition, knockdout of chTBK1 or chIRF7 results in IFN-β induced by chDDX3X overexpression was markedly decreased or disappear (Figure 6I). Based on the results of above, we concluded that chDDX3X could induce the expression of IFN-β via the chSTING-chTBK1-chIRF7-IFN-β signaling axis. This is the first study to report that DDX3X could activate IFN via the STING-mediated IFN pathway.

Birds have a smaller repertoire of immune genes than mammals (26). Chicken cells lack many key innate immune genes, such as RIG-I, TLR8, TLR9, and IRF3. Therefore, we conclude that there may be some differences in innate immunity in chicken cells. In our previous study, we found that a different RLR pathway is present in in RIG-I-null chicken cells (20).unlike mammalian STING, chicken STING can utilize MDA5 to recognize RNA viruses in RIG-I-null chicken cells, which is considered to be a mechanism that compensates for the lack of RIG-I in chickens. STING is a multifaceted IFN mediator. It has primarily been identified as a central IFN mediator in the progress of DNA recognition with the help of certain DNA sensors. Several lines of evidence suggest that mammalian STING is also involved in RNA recognition, which is mediated by RIG-I, but not MDA5 (27, 28). The discovery of the chDDX3X-chSTING-IFN-β signaling axis in chickens may provide new evidence for comparison of innate immunity characteristics between mammals and birds.

The absence of the RIG-I gene in chickens makes the RIG-I-STING-IFN-β signal pathway defective. DDX3X and RIG-I belong to the same DEAD family of RNA helicases, and they show certain similarities in structure and RNA recognition. In the above experiments, we demonstrated that chDDX3X may acts as a RNA virus sensor and can induce IFN-β production upon activation. However, we are not sure if the chDDX3X-chSTING-IFN-β pathway compensates for the lack of RIG-I, because it is yet unclear whether this pathway exists in mammals.

In summary, we identified chDDX3X as a chSTING-interacting protein and investigated its functions in the activation of IFN-β. We concluded that chDDX3X could respond to RNA- and RNA viruses and trigger IFN-β expression via the chDDX3X-chSTING-IFN-β signaling axis, which conferred a strong antiviral state against RNA viruses in chicken cells. These results help understand how IFN signaling is regulated in chickens and provides basic data about the general and individual characteristics of the innate immunity of avian and mammals.

## Author Contributions

JS, QN, and YC designed the experiment. QN and YC performed the experiments. YY and HW helped with the experiments. QN and YC wrote the paper.

### Conflict of Interest Statement

The authors declare that the research was conducted in the absence of any commercial or financial relationships that could be construed as a potential conflict of interest.

## Abbreviations

DDX3X: Asp-Glu-Ala-Asp polypeptide 3 X-linked
STING: stimulator of IFN genes
RIG-I: retinoic acid-inducible gene I
dsRNA: double-stranded RNA
TCID50: 50% tissue culture infective dose
AIV: avian influenza virus
ORF: open reading frame
aa: amino acid
MAVS: mitochondrial antiviral-signaling protein
MDA5: melanoma differentiation-associated gene 5
ISG: IFN-stimulated gene
poly(I:C): polyinosinic-polycytidylic acid
Co-IP: co-immunoprecipitatio.
